# Oncogenic Merkel Cell Polyomavirus T Antigen Truncating Mutations are Mediated by APOBEC3 Activity in Merkel Cell Carcinoma

**DOI:** 10.1158/2767-9764.CRC-22-0211

**Published:** 2022-11-04

**Authors:** Anni I. Soikkeli, Minna K. Kyläniemi, Harri Sihto, Jukka Alinikula

**Affiliations:** 1 Unit of Infection and Immunity, Institute of Biomedicine, University of Turku, Turku, Finland.; 2 Turku Doctoral Programme of Molecular Medicine, University of Turku, Turku, Finland.; 3 Turku Bioscience Centre, University of Turku and Åbo Akademi University, Turku, Finland.; 4 Department of Pathology, University of Helsinki, Helsinki, Finland.

## Abstract

Merkel cell carcinoma (MCC) is an aggressive skin cancer, which is frequently caused by Merkel cell polyomavirus (MCPyV). Mutations of MCPyV tumor (T) antigens are major pathologic events of virus-positive (MCPyV+) MCCs, but their source is unclear. Activation-induced cytidine deaminase (AID)/APOBEC family cytidine deaminases contribute to antiviral immunity by mutating viral genomes and are potential carcinogenic mutators. We studied the contribution of AID/APOBEC cytidine deaminases to MCPyV large T (LT) truncation events. The MCPyV *LT* area in MCCs was enriched with cytosine-targeting mutations, and a strong APOBEC3 mutation signature was observed in MCC sequences. *AICDA* and *APOBEC3* expression were detected in the Finnish MCC sample cohort, and *LT* expression correlated with *APOBEC3H* and *APOBEC3G*. Marginal but statistically significant somatic hypermutation targeting activity was detected in the MCPyV regulatory region. Our results suggest that APOBEC3 cytidine deaminases are a plausible cause of the *LT* truncating mutations in MCPyV+ MCC, while the role of AID in MCC carcinogenesis is unlikely.

**Significance:**

We uncover APOBEC3 mutation signature in MCPyV *LT* that reveals the likely cause of mutations underlying MCPyV+ MCC. We further reveal an expression pattern of APOBECs in a large Finnish MCC sample cohort. Thus, the findings presented here suggest a molecular mechanism underlying an aggressive carcinoma with poor prognosis.

## Introduction

Merkel cell carcinoma (MCC) is a rare but aggressive skin cancer mostly affecting the elderly and/or immunocompromised. The carcinoma has poor prognosis and at least two distinct etiologies. Approximately 80% of the tumors are caused by Merkel cell polyomavirus (MCPyV) where the virus is clonally integrated in the genome, have low mutation burden, and lack UV mutation signatures (1–7).Unlike virus-positive (MCPyV+) MCCs, virus-negative (MCPyV−) MCCs have high mutation burden and an UV mutation signature commonly seen in skin cancers (8–10). In addition to mutation accumulation, MCPyV+ and MCPyV− MCC differ in cell morphology and possibly the cell of origin (11, 12).

In MCPyV+ cancers, the MCPyV small tumor antigen (ST) and large tumor antigen (LT) cooperate to promote transformation and growth of the host cells (reviewed in ref. 13). The ST mediates its effect through interaction with various molecular complexes, which can reduce the activity of tumor suppressor p53, as well as stabilize and increase the expression of the LT (14–16).

MCPyV+ MCCs express highly mutated *LT* with substitutions and deletions frequently leading to premature stop codons and truncation of the protein (17). These mutations inactivate the C-terminal origin-binding domain and the helicase domain of the *LT* required for virus replication. Thus, the truncated LT is thought to enable stable integration of MCPyV and inhibit the DNA damage response caused by full-length replicated MCPyV genome (17, 18). The truncated LT retains the N-terminal LXCXE motif, which is required for the binding and inhibition of RB tumor suppressor protein along various other factors, thus enabling inappropriate cell-cycle progression and increase in cellular proliferation (19). While the truncating mutations of *LT* are central for the oncogenicity of MCPyV, the cause of these mutations is not known.

MCC was named after the cells it was presumed to arise from, the Merkel mechanoreceptor cells of the skin (20). Since then, it has been discovered that MCCs have a different protein expression pattern (21, 22), differentiation status (23), and location in the skin (24) compared with Merkel cells, calling the cancer-initiating cell type into question. In addition, MCPyV is unable to infect Merkel cells (25) and there are too few Merkel cells in the skin for them to be the cell of origin (26). Fibroblasts (12, 25), keratinocytes (12), and, interestingly, pro/pre-B cells (21) are proposed as the cell of origin of MCC but the issue remains unsettled.

MCPyV+ MCCs express the B-cell identity factor PAX5 and several immunoglobulin (Ig) classes (21, 27). MCCs express TDT, which is involved in antigen receptor gene recombination, and are shown to recombine their *Ig* genes, such as occurs in developing B cells (21). In addition, both MCPyV+ and MCPyV− MCCs are shown to express activation-induced cytidine deaminase (AID), a member of the AID/APOBEC family of cytidine deaminases (6, 28) AID is expressed almost exclusively in B cells, where it initiates somatic hypermutation (SHM) and class-switch recombination (CSR) in *Ig* genes. Being a powerful mutator, it is involved in the development of several cancer types, such as lymphoma, skin cancers (squamous cell carcinoma, basal-cell carcinoma, and melanoma), gastric cancers, and hepatocellular cancers. Particularly the members of APOBEC3 subfamily participate in antiviral defense (29, 30) and mutate viral genomes (31, 32), as well as contribute to carcinogenesis in, for instance, breast, cervical, and lung cancer (8, 29). APOBEC3A, APOBEC3B, and APOBEC3H are most tightly linked to carcinogenesis due to their frequent expression in cancers, nuclear localization, and ability to deaminate cytidines in genomic DNA (31, 33, 34). In addition, polyomaviruses have been shown to induce APOBEC3 expression (35, 36).

In *Ig* genes, AID-induced SHM is targeted to a 250–1,500 bp region downstream of the transcription start site, which codes for the variable domain of antibodies. SHM is targeted to the *Ig* loci by *Ig* enhancers and enhancer-like sequences (37). SHM is known to target several non-*Ig* genes outside the *Ig* locus, a phenomenon which is intimately linked to lymphoma (38–42).

We sought to investigate whether mutations in MCPyV *LT* could arise from an SHM-like cytidine deamination. We found APOBEC3 signature from *LT* area enriched in MCC sequences and *APOBEC3* expression in a Finnish MCC sample cohort. We found *AICDA* expression in a subset of Finnish MCC tumors and marginal SHM recruiting activity in the MCPyV regulatory region upstream of *LT* in a B-cell model, but the AID mutation signature was not enriched in *LT* sequences. We conclude that APOBEC3s, rather than AID, are mostly responsible for *LT* mutations in MCC.

## Materials and Methods

### Cloning Procedure

The sequences tested for SHM targeting activity were cloned to a GFP4-expressing vector either at a *Nhe*I/*Spe*I site (upstream position) or at a *Bam*HI site (downstream position). The tested sequences were first amplified from MCV-HF (RRID:Addgene_32057; ref. 43) using Q5 High-Fidelity DNA Polymerase (NEB). For downstream cloning, the GFP expression cassette was amplified from the GFP4 vector. Primers were designed according to In-Fusion primer design protocol. Cloning was performed with In-Fusion cloning (Takara) kit according to the manufacturer's protocol. The cloned plasmids were isolated with GeneJET Plasmid Miniprep Kit (Thermo Fisher Scientific), and the success of cloning was checked with restriction enzyme digestion. After successful cloning, new plasmid isolations were made with ZymoPure II Maxiprep kit (Zymo research).

### Transfection and Cell Culture

The chicken B-cell line DT40 (RRID:CVCL_0249) with modifications (*UNG*^−/−^*AICDA*^R/puro^; described in ref. 37) was received from David. G. Schatz. The cells were authenticated by testing their sensitivity for blasticidin and puromycin after targeted integration of the reporter construct, Western blotting of AID expression and functionally by carrying out the GFP loss assay with control reporters (Fig. 2B). The cells were cultured for approximately 25 days during the assay periods. The cells were cultured at +40°C, 5% CO_2_, 90% humidity. Growth media included RPMI1640 HEPES modification (Sigma) with 10% FBS (HyClone), 1% NCS (Biowest), 1x penicillin-streptomycin antibiotic (Gibco), 1x l-glutamine (Gibco), and 50 μmol/L β-mercaptoethanol. The cells were tested for lack of *Mycoplasma* on January 9, 2020 using PCR *Mycoplasma* test kit I/C (PromoCell).

The GFP loss assay was performed (as described in ref. 37). First, 12 × 10^6^ cells were transfected with 50 μg of *Not*I-linearized test sequences containing plasmids. For negative control, a plasmid including the GFP reporter without a test sequence was included. Transfection was performed with GenePulser electroporator (Bio-Rad) using settings 0.7 kV, 200 Ω, and 25 μF. Immediately after transfection, cells were transferred to 96-well plates to growth media with 5% extra FBS added. The day after transfection, blasticidin was added at a final concentration of 15 μg/mL to select the transfected cells. Cells were grown for approximately 7 days before primary clones were picked and tested for targeted integration using puromycin selection (final concentration 1 μg/mL). At least one targeted clone per test sequence was then subcloned by limiting dilution and cultured for 12 days before flow cytometry analysis.

### Flow Cytometry

Minimum of 12 independent subclones per tested sequence were analyzed using Accuri C6 cytometer (BD Biosciences). Results were then further analyzed with FlowJo software (RRID:SCR_008520) and GraphPad Prism 9 software (RRID:SCR_002798).

### Mutation Analysis

A total of 113 MCPyV *LT* sequences from MCC samples and 83 MCPyV *LT* sequences from healthy skin samples submitted to GenBank NCBI Virus database by the year 2020 were analyzed. The sequences varied in length between 65 and 2,885 bases. Sequences were aligned to the reference MCPyV isolate R17b (NCBI:txid 493803) sequence using SnapGene software (RRID:SCR_015052). All single-bp substitutions and insertion/deletion mutations relative to the MCPyV reference genome were included in the analysis. To avoid overrepresentation of MCPyV strain-specific variants, each mutation was calculated as one despite its potential occurrence in multiple sequences. The amount and distribution of single-bp substitutions as well as mutations leading to a stop codon were analyzed. WR**C**, T**C**W, and Y**C**C motifs and mutations affecting the underlined cytidines were calculated. These motifs were selected to represent AID, APOBEC3, and UV hotspots, as they are mutually exclusive and thus give a better resolution for mutation analysis and trinucleotide sites were used to achieve better comparability. Mutation frequency was calculated in 200 bp bins. The number of mutations was divided by the number of sequences analyzed. Because some analyzed sequences were truncated or otherwise partial, this bin-based approach prevented bias caused by different number of sequences obtained along the *LT* area. Graphs were made using GraphPad Prism 9 software.

### RNA Sequencing

RNA sequencing (RNA-seq) was performed with 82 RNA samples extracted from formalin-fixed, paraffin-embedded (FFPE) tumor tissue blocks. The samples are part of a larger set of tumor samples from a Finnish population-based sample series. RNA was extracted from two FFPE sections per primary MCC tumor using QIAGEN's QIAsymphony RNA extraction kit and QIAsymphony SP instrument. RNA concentration was measured prior to library preparation. Sequencing was performed in the sequencing unit of the Institute for Molecular Medicine Finland. RNA-seq libraries were prepared using QuantSeq 3′ mRNA-seq Library prep kit FWD (Lexogen) according to manufacturer's instructions and sequenced with Illumina HiSeq 2500 high output mode, v4 chemistry, and 2 × 100 bp reads. Raw read quality was analyzed with FastQC v0.11.8 (44) and MultiQC v1.7 (45). Raw reads were trimmed with BBDuk (BBMap version 38.87) according to instructions from library kit manufacturer. ERCC control sequences and MCPyV genome were combined to GRCh38 genome and trimmed reads were mapped to genome with STAR 2.7.2 (46) according to library kit manufacturer's instructions. Reads mapping to genes were counted with htseq-count using stranded mode (47). For counts per milion (CPM) normalization of read counts, the edgeR package v3.32.1 (48) was used. For annotations of read counts, R package org.Hs.eg.db v3.12.0 (49) was used. Analysis was performed in the CSC Puhti computing environment and in R version 4.5 in macOS 11.5.2. Fold changes of *AICDA* and *APOBEC*s were calculated by comparing normalized read counts of MCPyV+ and MCPyV− MCC samples to each other. Heatmap and similarity matrix were generated using Morpheus software provided by Broad Institute (RRID:SCR_017386).

### Statistical Analysis

Mann–Whitney *U* test was performed to evaluate statistical significance of the number of mutations, mutation distribution as well as the ratio and type of hotspot mutations between MCC and healthy control sequences. Mann–Whitney *U* test was also performed to evaluate statistical significance of the median GFP loss compared with GFP4 vector without test sequence and for comparison between upstream and downstream constructs. One minus the Spearman rank correlation was used as the calculation method for heatmap hierarchical clustering and Spearman rank correlation for similarity matrixes. Fisher exact test was used to calculate statistical differences of *AID/APOBEC*-expressing and nonexpressing MCC samples. Fisher exact test was also used to calculate statistical differences of high and low sun-exposed MCC tumors and their MCPyV status. Statistical analyzes were performed using GraphPad Prism 9 software.

### Data Availability Statement

The RNA-seq data are available under the BioProject PRJNA775071 in NCBI Sequence Read Archive (SRA) database.

## Results

### MCPyV *LT* is Enriched in Cytosine-targeting Mutations in MCC

**FIGURE 1:**
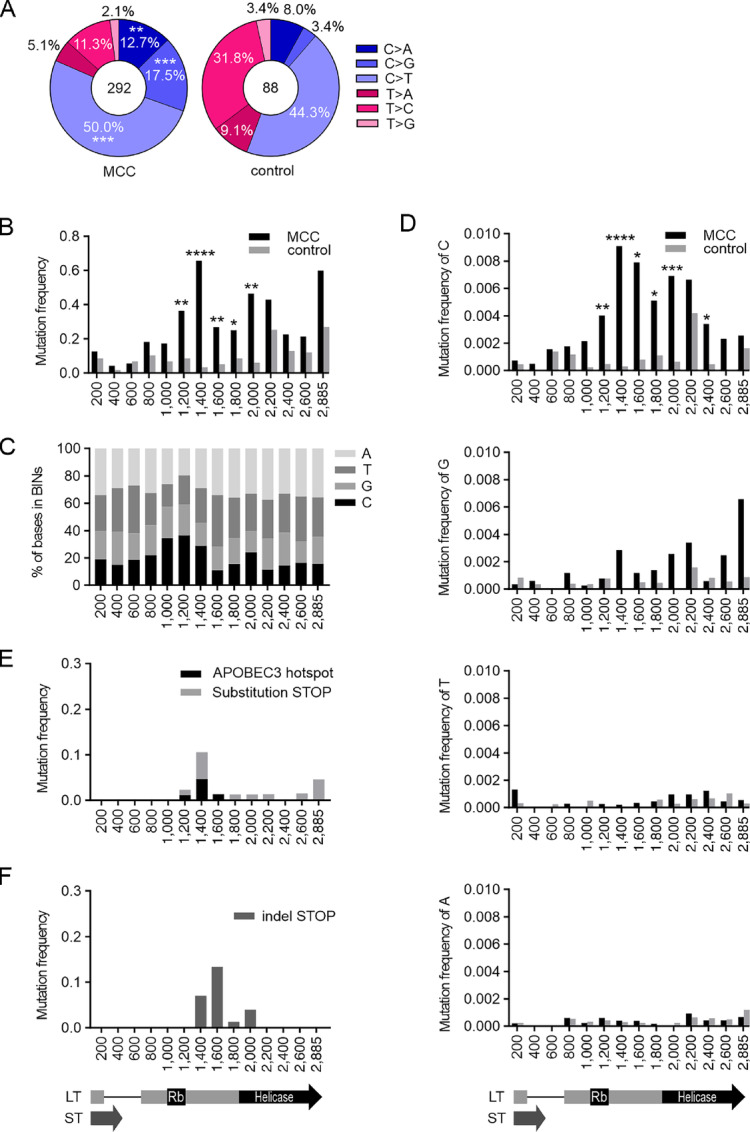
Mutations in MCV *LT* area in MCC and control samples. **A,** Substitution mutations in *LT* area in MCC and control samples in six mutation classes: C>A, C>T, C>G and T>A, T>C, T>G. Number of analyzed substitutions is shown in the middle. **B,** Distribution of substitutions along *LT* area is in MCC and control samples. *LT* area is divided to 200 bp bins (final bin 285 bp) and mutation frequency (number of mutations/number of sequences per bin) is shown. A schematic of *ST* and *LT* area is depicted below the graphs. **C,** Base content in *LT* area in each bin. **D,** Distribution of substitutions by mutated base in each bin. **E,** Distribution of in-frame stop codons introduced by substitution mutations in each bin (light gray). Black columns represent the proportion of in-frame stop codons that reside within APOBEC3 TCW hotspots. **F,** Distribution of in-frame stop codons in each bin introduced by insertion/deletion (indel) mutations before or in the indicated bin. Statistical significances between MCC and control mutations are determined using Mann–Whitney *U* test. **** <0.0001, *** <0.001, ** <0.01, * <0.05.

We compared substitution and insertion/deletion mutations in 83 sequenced healthy skin samples and 113 sequences from published MCC samples. Of the 318 mutations in the MCC samples, 292 (91.8%) were substitutions and 26 (8.2%) were insertions/deletions, while of the 90 mutations in the control samples, 88 were substitutions (88%) and two insertions/deletions (12%; Table 1). The substitutions occurred mostly at cytosine bases: 238 (81.5%) in the MCC and 49 (55.7%) in the control samples (Fig. 1A).

**TABLE 1:** Summary of mutations detected in *LT* area

*LT* area	All	*P*	subst	*P*	indel	*P*	No. of seq	No. of seq bases
MCC mutations	318	<0.0001	292	<0.0001	26	0.0002	113	206646
MCC in-frame STOP	39	<0.0001	19	0.0001	20	<0.0001	65	
Control mutations	90		88		2		83	172425
Control in-frame STOP	0		0		0		0	

To determine the distribution of the mutations in the MCPyV T antigen area, we divided the mutations into 200 bp bins by normalizing the number of mutations in each bin area to the number of sequences covering the entire bin. In the control samples, the mutations had a relatively even distribution along the T antigen area (Fig. 1B), with some concentration toward the end of the *LT* gene (peaking at bins 2,200 bp and 2,885 bp). In contrast, the mutation frequency in MCC samples increased after 1,000 bp (bins ending at 1,200 bp, *P* = 0.0089; 1,400 bp, *P* < 0.0001; 1,600 bp, *P* = 0.0088; 1,800 bp, *P* = 0.0370; 2,000 bp, *P* = 0.0014). Because the bins 1,000–1,400 have more cytidines than the rest of the *LT* area (Fig. 1C), we normalized the number of substitutions to the number of given bases in each bin (Fig. 1D). This reveals that most of the substitutions enriched in MCC samples have indeed occurred at cytidines (bins ending at 1,200 bp, *P* = 0.0036; 1,400 bp, *P* < 0.0001; 1,600 bp, *P* = 0.0207; 1,800 bp, *P* = 0.0363; 2,000 bp, *P* = 0.0007; 2,400 bp, *P* = 0.0494) and that the cytidines account for most of the mutations between RB and helicase domains (bins ending at 1,200–2,000 bps).

Given that a truncation mutation in *LT* is a major oncogenic event in MCPyV+ MCC (18) and the fact that a substitution of a C is a likely way to generate a stop codon due to their lack of cytidines, we considered whether the large number of substitutions from C in MCC samples is a result of positive selection of stop codons.

While most (75.7%) of the substitutions targeting Cs were in the region spanning 1,200–2,200 bp (Fig. 1D), 6.5% of all substitutions in the T antigen area caused an in-frame stop codon (Table 1), and the in-frame stop codon distribution (Fig. 1E) explains only a very small proportion of the overall distribution of mutations in MCC samples (Fig. 1B). Instead, out of all insertion and deletion mutations, 77% caused an in-frame stop codon, all of which were in the 1,200–2,200 bp region (Table 1; Fig. 1F). Overall, 57.5% of the MCC sequences carried an in-frame stop codon. Many of the sequences analyzed here were partial, hence the amount of in-frame stop codons detected is likely an underestimation. Nevertheless, these findings recapitulate the previously described increase in premature stop codons in the *LT* (17, 23, 50), but do not explain the bias towards mutations at Cs. It is conceivable that the C bias arises from the activity of AID/APOBEC family of cytidine deaminases.

### MCPyV Regulatory Region Shows Marginal SHM Recruiting Activity

**FIGURE 2:**
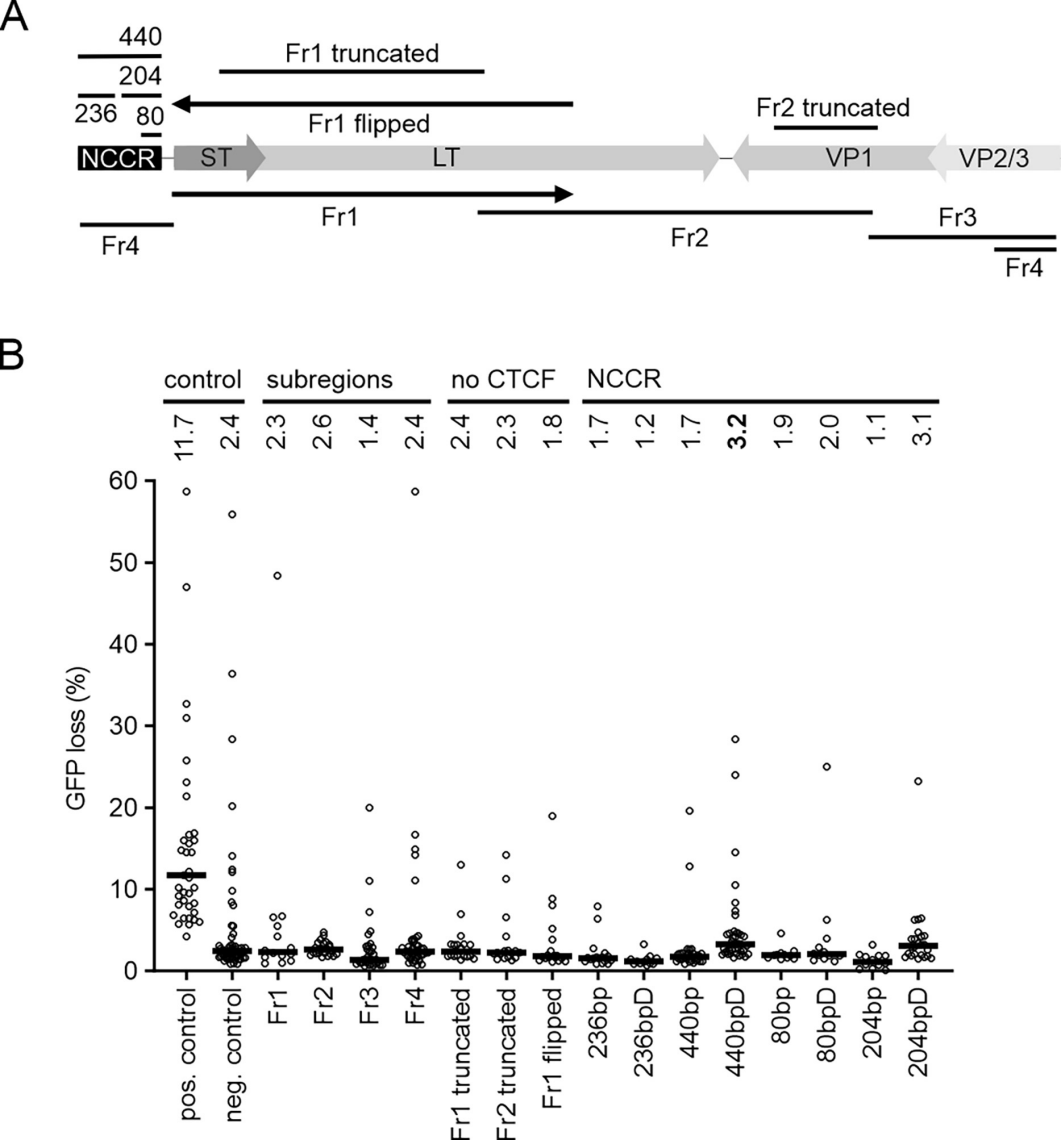
SHM targeting activity of MCV genome. **A,** Schematic of MCPyV genome and fragments tested in GFP loss assay. Arrows in “Fr1” and “Fr1 flipped” highlight the opposite orientation of these fragments relative to the GFP reporter (37). **B,** SHM targeting activity in MCPyV genome as measured in GFP loss assay. Four subregions (Fr1, Fr2, Fr3, and Fr4) of MCPyV genome as well as truncated Fr1, Fr2, and Fr1 in reverse orientation (Fr1 flipped) were tested (no CTCF). MCPyV NCCR (440 bp) and its 236, 80, and 204 bp subfragments were tested also downstream of the transcription unit (236bpD, 440bpD, 80bpD, and 204bpD). Human Ig lambda enhancer core (37) was used as positive control and the reporter without a test sequence was used as negative control. Median values are shown. Ten datapoints are outside of axis limits. Mann–Whitney *U* test was used to determine statistical significance. *P* values: pos. control <0.0001; Fr1 0.7863; Fr 2 0.4659; Fr3 0.0013; Fr4 0.7144; Fr1 truncated 0.9215; Fr2 truncated 0.7978; MCV Fr1 flipped 0.0901; 236bp 0.0009; 236bpD <0.0001; 440bp 0.0004; 440bpD 0.0161; 80bp 0.0672; 80bpD 0.4128; 204bp 0.0001; 204bpD 0.3783.

Because of reported AID expression in MCC (6, 28), we wanted to test the role of SHM in causing mutations of the MCPyV *LT*. We tested the possibility of the MCPyV genome having *cis*-acting SHM recruiting sequences similar to *Ig* loci. To do this, we performed a GFP-loss assay in DT40 B cells, which measures SHM recruitment activity (37). We first tested four partly overlapping regions of the MCPyV genome (Fr1, Fr2, Fr3, and Fr4; Fig. 2A). None of these regions showed significant SHM recruitment activity (Fig. 2B). SHM activity in the genome is restricted within topologically associating domains, whose boundaries may prevent the spreading of SHM from one domain to the next (51). Thus, any insulator, such as a CCCTC-binding factor (CTCF) binding site in the test DNA fragment between the potential active SHM-recruiting region and the *GFP* reporter gene, could prevent GFP loss. To address this, we tested subregions of Fr1 and Fr2 that do not contain CTCF sequences (Fr1 truncated and Fr2 truncated, respectively) and tested Fr1 in reverse orientation, which moves the CTCF site away from between the fragment core and the GFP transcription unit of the reporter (Fr1 flipped). None of these modified fragments exhibited SHM targeting activity (Fig. 2B).

Because SHM targeting elements in *Ig* loci are found in regulatory regions (enhancers), we also tested the MCPyV noncoding control region (NCCR) more carefully (Fig. 2). We tested NCCR fragments both upstream and downstream of the GFP expression cassette, as positioning the element upstream of the reporter affects the transcriptional output but not when positioned downstream (52). Interestingly, the downstream position of the 440 bp fragment (440bpD) showed low but statistically significant (3.2% GFP loss, *P* = 0.0161) SHM targeting activity (Fig. 2B). Further splitting the 440 bp fragment into smaller 80 and 204 bp fragments reduced the targeting activity below detection limit, but similarly to the 440 bp fragment, the 204 bp fragment had more SHM targeting activity in downstream than in upstream position (440bpD: 3.2% and 440bp 1.7%, *P* < 0.0001; 204bpD 3.1% and 204bp 1.1%, *P* < 0.0001). Therefore, we conclude that the NCCR of MCPyV can marginally recruit SHM to a neighboring transcription unit in cells that are capable of SHM.

### Mutations in MCC *LT* Area Concentrate to APOBEC3 Hotspots

**FIGURE 3:**
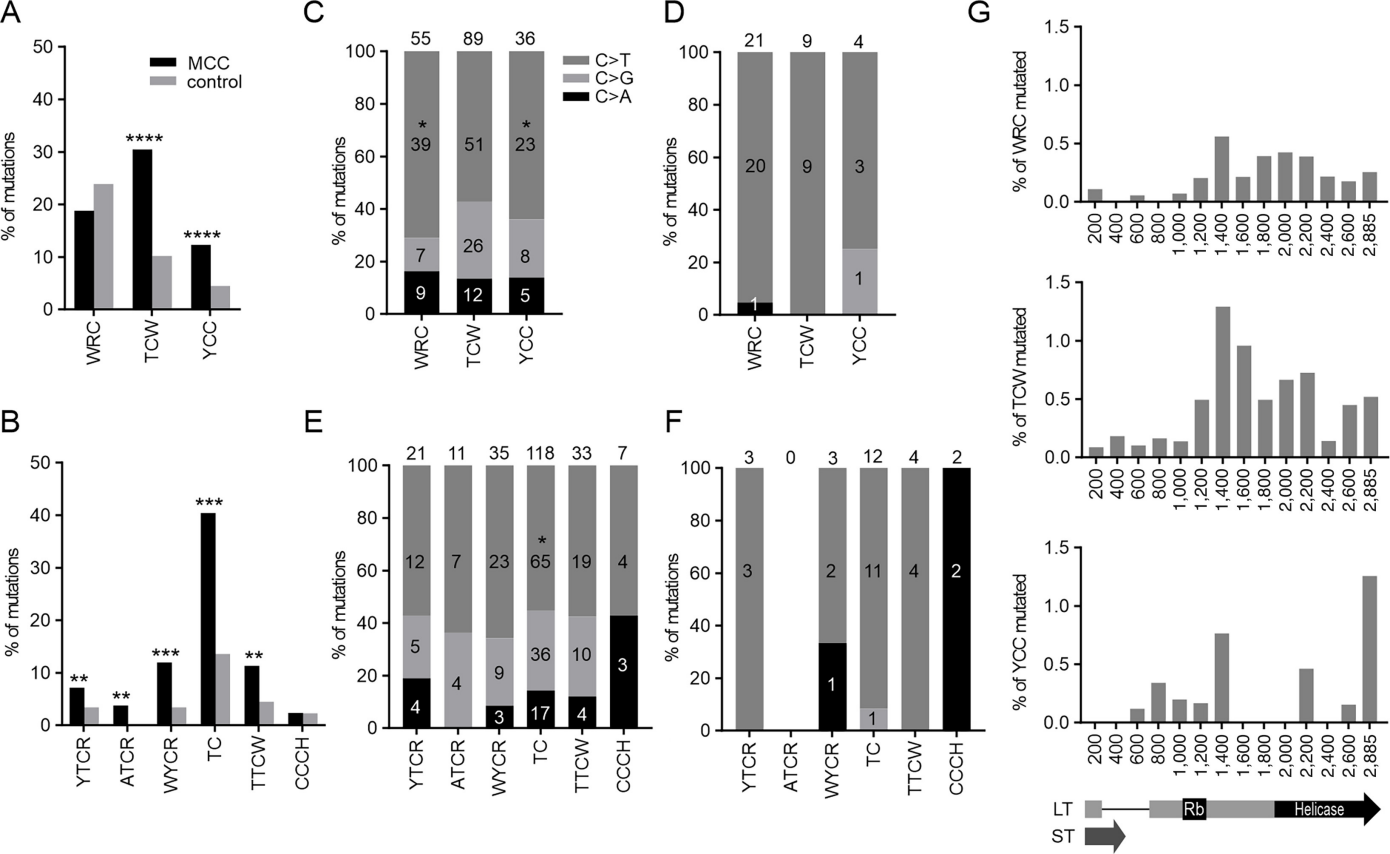
AID, APOBEC, and UV hotspot mutations in *LT* area. **A,** The proportion of substitutions in AID (WRC), APOBEC3 (TCW), and UV (YCC) hotspots in sequences from MCC (black) and control (gray) samples. **B,** Proportion of APOBEC3 subfamily hotspot mutations in YTCR, ATCR, WYCR, TC, TTCW, and CCCH in MCC samples and control samples. Colors as in **A**. **C,** The proportion of mutation type (C>T dark gray, C>G light gray and C>A black) in each hotspot in MCC samples. Number of each mutation type as well as total number of mutations in hotspots is indicated. **D,** The proportion of mutation type (C>T, C>G, and C>A) in each hotspot in control samples. Numbers and colors are as in **C**. **E,** The proportion of mutation type (C>T, C>G, and C>A) in each hotspot in MCC samples. Numbers and colors are as in **C**. **F,** The proportion of mutation type (C>T, C>G, and C>A) in each hotspot in control samples. Numbers and colors are as in **C**. **G,** Distribution of mutated WRC, TCW, and YCC hotspots along MCPyV *LT* area. Statistical significances between MCC and control hotspot mutations and their types are determined using Mann–Whitney *U* test. **** <0.0001, *** <0.001, ** <0.01, * <0.05.

To explore the role of AID/APOBEC deaminases in *LT* mutations, we analyzed the immediate sequence context of *LT* substitution mutations for enrichment of AID hotspot sequences (WR**C**). We found that while 23.9% of mutations were in WR**C** hotspots in control samples, they were not enriched in MCC samples (18.0%, *P* = 0.0630; Fig. 3A).

As the mutations were clearly accumulated in C bases (Fig. 1), and cytidine deaminase APOBEC3 signature was recently established in MCPyV in the context of T**C** dinucleotides (36), we analyzed APOBEC3 hotspot mutations in *LT* in the T**C**W trinucleotide context. The proportion of mutations at APOBEC3 hotspots was increased 3-fold in MCC samples (30.5%) compared with healthy controls (10.2%, *P* < 0.0001; Fig. 3A). Interestingly, 42.9% of the in-frame stop codons caused by substitution mutations at Cs were also APOBEC3 hotspot mutations, out of which 60% were between RB and helicase domains (Fig. 1F). In contrast, none of the in-frame stop codons after this region were in APOBEC3 hotspots.

Individual APOBEC3 enzymes have slightly different specificities in addition to the general T**C**W motif (30). APOBEC3A prefers YT**C**R, APOBEC3B AT**C**R, APOBEC3C WY**C**R, APOBEC3D/H T**C**, APOBEC3F TT**C**W, and APOBEC3G CC**C**H (30). Therefore, we analyzed APOBEC3A, APOBEC3B, APOBEC3C, APOBECD/H, APOBEC3F, and APOBEC3G-hotspot mutations in more detail (Fig. 3B). Out of these hotspot motifs, we saw a proportional increase in APOBEC3A (2.1-fold, *P* = 0.0019), APOBEC3B (*P* = 0.0056), APOBEC3C (3.5-fold, *P* = 0.0005), APOBEC3D/H (3.0-fold, *P* = 0.0001), and APOBEC3F (2.5-fold, *P* = 0.0025) hotspot mutations (Fig. 3B). Mutations were not increased in APOBEC3G CC**C**H hotspots (*P* = 0.3066). However, there is considerable overlap between some of these hotspots, and mutations induced by the AID/APOBEC family are not strictly restricted to these motifs (29).

UV radiation is also a probable source of C mutations in skin cancers, and UV mutation patterns are observed in MCPyV− MCCs, but not in MCPyV+ MCCs (7). UV radiation causes frequent C>T and CC>TT mutations at dipyrimidine sites (53, 54). Because T**C**C or C**C**C trinucleotides are mutated most frequently in the single-base mutation signatures SBS7a and SBS7b of the COSMIC database, we used Y**C**C here as a UV hotspot motif. The difference in the proportion of mutations in Y**C**C was 2.7-fold in healthy versus MCC samples (4.5% and 12.3%, respectively, *P* < 0.0001), but these mutations were infrequent (Fig. 3A). There were only three double-base substitutions at CC dinucleotides in the MCC samples: CC>AT, CC>GT, and GG>AA. It should be noted that using Y**C**C as UV radiation hotspot may underestimate the number of UV-induced mutations. Using tumor location information combined to the viral status of the Finnish MCC samples (assessed by qPCR; refs. 55, 56), we determined that 35.6% of MCPyV+ MCCs appear in areas of body where low sun exposure is expected, whereas only 20.6% of MCPyV− MCCs appear at low sun-exposed areas. However, the difference was not statistically significant (Fisher exact test, *P* = 0.1320).

Analysis of the proportions of C>A, C>G, and C>T substitutions at each hotspot (Fig. 3C–F) showed that in the context of WRC and YCC hotspots, C>T substitutions were predominant, while in APOBEC3 hotspots also C>G substitutions were common. This is in line with the mutation type distribution described for AID, APOBEC, and UV radiation (10, 29, 39, 42) and further implicates these factors as mutators in MCC. Note, that these classifications are not definitive, as several factors influence which base is incorporated to the site of initial lesion.

Curiously, AID and APOBEC3 hotspots were mainly mutated after RB binding site (starting from 1,200 bp bin), whereas UV hotspot mutations seemed to have accumulated in the beginning of the *LT* area (up until 1,400 bp bin) and at the very end of the *LT* (at 2885 bp bin; Fig. 3G). Overall, APOBEC3 had the highest hotspot mutation rate and the best resemblance with overall C-mutation distribution, enforcing the notion of APOBEC3 signature in the *LT*.

### Finnish MCCs Express *AICDA* and *APOBEC3*s

To investigate the expression of AID/APOBEC family proteins in MCC tumors, we analyzed the expression levels of *APOBEC*s and *AICDA*, the gene encoding for AID, from 3´Tag RNA-seq data of 82 Finnish MCC tumors. 61.0% of the MCC samples expressed MCPyV *LT*, while 39.0% had undetectable levels of *LT* expression in this sample cohort. On the basis of *LT* expression, the tumors were divided into MCPyV+ and MCPyV− MCCs to detect whether the expression of *AID/APOBEC* family members is linked to *LT* expression. The 3′ RNA-seq probably underestimates the number of MCPyV+ MCC samples, as in previous studies using other methods to analyze the same cohort, the number of MCPyV+ MCC samples was higher (27, 55, 57).

*AICDA* expression levels were 1.4-fold higher, and the proportion of *AICDA*-expressing samples was higher in the MCPyV− group (34.4%) than in the MCPyV+ group (32.0%; Table 2).

**TABLE 2:** Percentage of *AID/APOBEC* expressing samples and fold change of RNA expression level in MCPyV+ and MCPyV− groups

	*AICDA*	*APOBEC1*	*APOBEC2*	*APOBEC3A*	*APOBEC3B*	*APOBEC3C*	*APOBEC3D*	*APOBEC3F*	*APOBEC3G*	*APOBEC3H*	*APOBEC4*
MCPyV+	32.0	0.0	14.0	46.0	2.0	94.0	20.0	76.0	92.0	42.0	4.0
MCPyV−	34.4	3.1	21.9	37.5	6.3	96.9	18.8	90.6	93.8	9.4	3.1
*P*	>0.9999	0.3902	0.3812	0.4984	0.5574	>0.9999	>0.9999	0.1432	>0.9999	*0.0024*	>0.9999
Fold change MCPyV+/ MCPyV−	0.7	0.0	0.3	1.2	0.2	1.2	3.4	0.8	1.5	4.0	1.7

*APOBEC1* and *APOBEC4* were expressed sparsely (Table 2), and their expression levels were very low. *APOBEC2* levels were higher (3.1-fold), and *APOBEC2* was more frequently expressed in the MCPyV− group than in the MCPyV+ group (21.9% and 14.0, respectively). However, neither APOBEC2 nor APOBEC4 are known to have deaminase activity (29).

**FIGURE 4:**
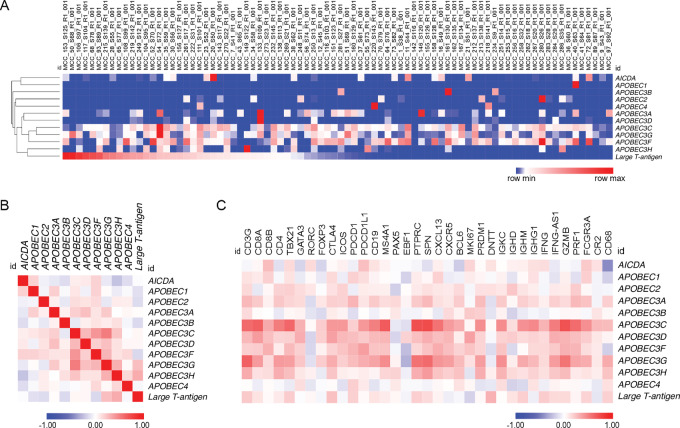
*AICDA* and *APOBEC* expression in Finnish MCC sample cohort. **A,** Heatmap of *AID/APOBEC* expression across individual tumors. Hierarchical clustering was performed with one minus Spearman rank correlation method. MCC samples are arranged according to *LT* expression level. **B,** Similarity matrix of *AICDA*, *APOBEC,* and *LT* expression. *APOBEC3H* and *APOBEC3G* are significantly coexpressed with *LT* (Spearman rank correlation *APOBEC3H r* = 0.32, *P* = 0.004 and *APOBEC3G r* = 0.25, *P* = 0.025). **C,** Similarity matrix of selected *T lymphocyte*, *B lymphocyte*, *NK-cell*, and *follicular dendritic cell markers* and their coexpression with *AICDA*, *APOBEC*s, and *LT*.

More tumors in the MCPyV+ group expressed *APOBEC3A* (46.0% vs. 37.5%), *APOBEC3D* (20.0% vs. 18.8%), and *APOBEC3H* (42.0% vs. 9.4%) than in the MCPyV− group, and their mRNA expression of was stronger (1.2-fold, 3.4-fold, and 4.0-fold, respectively; Fig. 4A). The majority of tumors expressed *APOBEC3C*, *APOBEC3F,* and *APOBEC3G,* and the expression of *APOBEC3C* and *APOBEC3G* was increased in MCPyV+ tumors (1.2- and 1.5-fold, respectively). A very small percentage of all MCCs in this sample cohort expressed *APOBEC3B*.

We performed hierarchical clustering for *AID/APOBEC* and *LT* expression data (Fig. 4A). The *LT* expression clustered together with *APOBEC3* subfamily, excluding *APOBEC3B*. *APOBEC3H,* and *APOBEC3G* expression correlated with *LT* expression (Fig. 4B; Spearman correlation *APOBEC3H r* = 0.32, *P* = 0.004; *APOBEC3G r* = 0.25, *P* = 0.025), suggesting a possible mechanistic link between MCPyV infection and *APOBEC3G/APOBEC3H* expression.

*APOBEC3B* excluded, *APOBEC3*s and *LT* coexpressed with markers of T lymphocytes (*CD8A, CD8B, CD4, CD3G, and granzyme B;*Fig. 4C). Also, IFNγ (*IFNG*), and IFNG regulating long noncoding RNA (*IFNG-AS*) coexpressed with these *APOBEC3*s. This is in line with the presence and potential contribution of tumor-infiltrating lymphocytes for *APOBEC3* expression in MCC tumors (36, 58). Interestingly, we found coexpression of B-lymphocyte markers (*CD19* and *MS4A1*, encoding for CD20) with most of these *APOBEC3*s. This suggests that as well as T lymphocytes, B lymphocytes also infiltrate MCC tumors.

In addition, germinal center B-cell markers (*BCL6*, *Ki67*, *CD4*, *ICOS*, and *PDCD1* encoding for PD-1) were expressed, which could indicate formation of tertiary lymphoid structures. Therefore, we cannot exclude tumor-infiltrating B lymphocytes rather than malignant cells as the cellular source of AID expression in the tumors.

Overall, our findings indicate clear APOBEC3 signature and *APOBEC3* expression in MCPyV+ MCC tumors.

## Discussion

The human AID/APOBEC gene family of cytosine deaminases codes for 11 proteins with various functions: AID, APOBEC1, APOBEC2, APOBEC3A, APOBEC3B, APOBEC3C, APOBEC3D, APOBEC3F, APOBEC3G, APOBEC3H, and APOBEC4 (refs. 29, 59; reviewed in ref. 60). APOBEC2 and APOBEC4 do not appear to catalyze cytosine deamination causing genomic mutations and AID drives SHM and CSR by deaminating single-stranded cytidines in *Ig*. The 7-member APOBEC3 subfamily contributes to restriction of viral infections with both deaminase-independent mechanisms as well as through cytosine deamination (60). APOBEC3G was first found in defense against HIV-1, where virion-packed APOBEC3s can introduce G-to A-mutations, viral DNA degradation, and lethal coding mutations of the genome-integrated virus (61). In addition to HIV-1, APOBECs are associated in defense against several cancer-associated viruses such as human papilloma virus, hepatitis B virus, and human T lymphotropic virus 1 (60, 62). APOBEC3s have low expression in a range of host cell types, but their expression is greatly increased upon viral infection and cytokine-induced stimuli. Not surprisingly, APOBECs and viruses have been engaged in an arms race of immune evasion evident in polymorphism and copy-number variation in *APOBEC3* gene as well as selective pressure on viral genomes sequences, which can be seen as a “footprint” in the genomes of a wide range of viral species, including *Polyomaviridae* (63).

While beneficial for antiviral immunity, the AID/APOBEC are known to induce genomic mutations and chromosomal aberrations with oncogenic outcomes in lymphocytes as well as in solid tissue cancers (reviewed in ref. 29). However, the contribution of APOBECs in cancer via mutations of a viral genome has remained poorly understood.

In this study, we found APOBEC3 deaminase mutation signature in the MCC *LT* area. Up to 43% of in-frame stop codon-causing substitutions occurred in APOBEC3 hotspots with the mutational outcome expected for APOBEC3s, and *APOBEC3* was widely expressed in MCPyV+ MCC tumors. Thus, our analysis implicates APOBEC3s as the primary mutators of *LT*.

APOBEC3s are largely agreed to target T**C**W motifs (29, 64). Focusing the analysis on this hotspot avoids mistakenly counting in UV-induced mutations, which frequently target pyrimidine dimers (54). CC>TT substitutions were absent in *LT*, and 35% of the Finnish MCPyV+ MCC tumors were in low sun exposure areas of the body. Thus, our findings do not support the role of UV in *LT* mutations.

Using a recently specified target motif preference for individual APOBEC3 subfamily members (30), we could not discriminate a specific APOBEC3 as the primary mutator in the *LT*. As expected, due to a loose definition and overlap with other hotspots, the T**C** dinucleotides suggested as APOBEC3D/H hotspot (30) were most frequently mutated, agreeing with the conclusion drawn from characterizing mutations in MCPyV by using the T**C** dinucleotide as APOBEC3 hotspot (36).

MCPyV can induce APOBEC3A, APOBEC3B, and APOBEC3G expression in cell models (35, 36), where at least APOBEC3B can be upregulated by IFNγ (36). APOBEC3B has been the primary suspect out of the APOBEC family in cancers (65), but its expression was very rare in our relatively large MCC tumor dataset and did not significantly correlate with *LT* expression. Nevertheless, the host cell may have expressed *APOBEC3B* during initial MCPyV infection or earlier in tumor development before the tumor removal, because APOBEC expression is naturally episodic and fluctuating (66). Thus, the involvement of APOBEC3B cannot be entirely excluded. Nevertheless, we found coexpression of other *APOBEC3*s with *INFG* and *INFG* regulating noncoding RNA and our findings are in line with the INFγ-induced APOBEC3 expression.

In contrast to *APOBEC3B*, the expression of *APOBEC3A* and *APOBEC3H* was widespread. There is accumulating evidence that APOBEC3A and 3H are equally or even more potent mutators in cancers than APOBEC3B (67–69). *APOBEC3A* and *APOBEC3H* were expressed in a larger proportion of Finnish MCPyV+ MCC samples (46.0% and 42.0%, respectively) than MCPyV− MCC samples (37.5% and 9.0%, respectively). The difference was statistically significant for *APOBEC3H*, which also showed statistically significant coexpression with *LT*. In addition, specific hotspot mutations (at YT**C**R and T**C**) were frequent, and also both APOBEC3A and APOBEC3H can mutate T**C**W (67–69). It should be noted that the mutation pattern and RNA expression data in our study came from different sources, making it possible, although unlikely, that the different genetic background of the subjects obscured our findings.

*APOBEC3C*, *APOBEC3F*, and *APOBEC3G* were expressed in the majority of the MCC samples. *APOBEC3C* and *APOBEC3G* levels were higher in MCPyV+ MCCs compared with MCPyV− MCCs, and *APOBEC3G* showed statistically significant coexpression with *LT*, which could implicate an immune response to MCPyV infection and make APOBEC3C and APOBEC3G potential *LT* mutators. Because *APOBEC3D* was expressed only marginally, and along with APOBEC3G and APOBEC3F, mainly reside in the cytoplasm (59), our data do not strongly support them as primary mutators.

Given the contribution of APOBEC3s in genomic cancer mutations and viral mutations in antiviral defense, our findings strongly suggest that APOBEC3 subfamily, particularly APOBEC3A, APOBEC3H, APOBEC3C, and to smaller extent APOBEC3G, contribute to the *LT* mutation signature and potentially premature stop codon formation.

AID is expressed in activated B lymphocytes and its association in B-lymphocyte cancers is well established (70) and has been implicated in skin cancers such as melanoma (71). We also detected *AICDA* expression in a subset of Finnish MCC samples, where the expression was stronger and slightly more frequently expressed in MCPyV− MCC samples, conforming with a previous finding (6). We also did not find AID mutation signatures enriched in MCC samples over control.

We found very weak, yet statistically significant, SHM targeting activity in the MCPyV NCCR. Therefore, it is possible that the SHM targeting activity possessed by the MCPyV NCCR could target AID-mediated SHM to the adjacent T antigen area (or other nearby genes), similar to the enhancers in *Ig* loci. As SHM is a property of germinal-center B lymphocytes, the scenario of AID inducing carcinogenic mutations in MCPyV is more likely if MCC indeed derived from activated skin-resident B lymphocytes. Expression of B-lymphocyte markers in MCC (reviewed in 72) fit better for pro/pre-B lymphocyte stage. Thus, we conclude that AID-induced SHM is unlikely the primary mechanism for *LT* mutations, but its role in MCC carcinogenesis cannot be entirely excluded. Because there is a correlation between APOBEC mutations and DNA replication (29) and the NCRR also has the replication of origin for the MCPyV, it is conceivable that the mutations causing the GFP loss in our reporter system result from APOBEC activity in the DT40 B cells used to carry out the assay. This remains to be addressed experimentally.

Tumors are often infiltrated with immune cells (73), and T lymphocytes, macrophages, and natural killer cells were detected more frequently in MCPyV+ MCCs than in MCPyV− MCCs, demonstrating immune cell infiltration also in MCC (58). The role of B lymphocytes in tumor environment has not been studied as extensively as other immune cells, but there is evidence showing the importance of tumor-infiltrating B lymphocytes (74). We did observe expression of B-cell markers including *AICDA* in addition to T-lymphocyte markers in the RNA-seq data. It remains to be investigated whether a high number of tumor-infiltrating B lymphocytes could also explain the B-lymphocyte marker expression in MCC.

In conclusion, our findings support the view where APOBEC3 enzymes mutate MCPyV *LT* area and that this mutational activity is a major cause of premature stop codon formation and thus MCC carcinogenesis.
